# Pd Nanoparticles and MOFs Synergistically Hybridized Halloysite Nanotubes for Hydrogen Storage

**DOI:** 10.1186/s11671-017-2000-5

**Published:** 2017-03-31

**Authors:** Jiao Jin, Jing Ouyang, Huaming Yang

**Affiliations:** 1 Centre for Mineral Materials, School of Minerals Processing and Bioengineering, Central South University, Changsha, 410083 China; 2 Hunan Key Lab of Mineral Materials and Application, Central South University, Changsha, 410083 China; 3 State Key Lab of Powder Metallurgy, Central South University, Changsha, 410083 China

**Keywords:** Pd nanoparticles, Metal–organic frameworks (MOFs), Halloysite nanotubes, Hybrid, Hydrogen storage

## Abstract

Natural halloysite nanotubes (HNTs) were hybridized with metal–organic frameworks (MOFs) to prepare novel composites. MOFs were transformed into carbon by carbonization calcination, and palladium (Pd) nanoparticles were introduced to build an emerging ternary compound system for hydrogen adsorption. The hydrogen adsorption capacities of HNT-MOF composites were 0.23 and 0.24 wt%, while those of carbonized products were 0.24 and 0.27 wt% at 25 °C and 2.65 MPa, respectively. Al-based samples showed higher hydrogen adsorption capacities than Zn-based samples on account of different selectivity between metal and hydrogen and approximate porous characteristics. More pore structures are generated by the carbonization reaction from metal–organic frameworks into carbon; high specific surface area, uniform pore size, and large pore volume benefited the hydrogen adsorption ability of composites. Moreover, it was also possible to promote hydrogen adsorption capacity by incorporating Pd. The hydrogen adsorption capacity of ternary compound, Pd-C-H3-MOFs(Al), reached 0.32 wt% at 25 °C and 2.65 MPa. Dissociation was assumed to take place on the Pd particles, then atomic and molecule hydrogen spilled over to the structure of carboxylated HNTs, MOFs, and the carbon products for enhancing the hydrogen adsorption capacity.

## Background

Environmental crisis of fossil fuels and their predicted exhaustion are global concerns. Hydrogen has been considered as one of the most promising substitutes for the fossil fuels for the advantages of sustainable, renewable energy and abundant energy supply with the additional benefits of potentially allowing the production of zero-emission vehicles. However, the commercialization of hydrogen has been limited by the lack of a safe and efficient hydrogen storage system at room temperature [1]. Traditional gas compression and cryogenic liquid technologies faced the problems of high cost, low energy efficiency, and high requirements for the equipments. Meanwhile, most of materials for hydrogen storage suffered from these drawbacks of poor adsorption/desorption reversibility, inherent slow kinetics, thermodynamic energy inefficiency, secondary pollution caused by the reaction products, and high costs of production and regeneration [2, 3]. In recent years, sorbent approaches of hydrogen storage represented one potential strategy for effective and relatively safe hydrogen storage [4, 5]. Hydrogen storage on solid substrates by physisorption was attractive mainly because of its flexibility in enabling subsequent release of the adsorbed gas, if required [6]. In particular, metal–organic frameworks (MOFs) have attracted much attention for applications in adsorption and separation processes due to their high specific surface area and microporosity [7–9]. Natural halloysite nanotubes (HNTs) feature exceptional properties, such as relatively high specific surface area, high porosity, high cation-exchange capacity, low cost, and long life cycle [10–12], which make HNTs a perfect candidate for hydrogen storage as natural mineral [13, 14]. Mineral composite materials have plenty of excellent properties [15–19]. However, these adsorbed materials were not sufficiently effective to represent the final solution of hydrogen storage [1, 6, 20–22]. The basic idea was to find appropriate metals to modify the adsorbed materials aiming at enhancing hydrogen adsorption which proceeds via a spillover mechanism [23–30]. Spillover has been defined as the transport of an active species adsorbed on one site to another site that would not typically adsorb the active species at the prevailing conditions. Hydrogen storage by spillover has been a promising approach to enhance the hydrogen storage capacities in nanostructured materials including carbon nanomaterials, zeolites, mineral materials, porous materials, and metal–organic frameworks [13, 30–38].

The present study aimed to investigate an appropriate method to synthesize novel composites of HNTs and MOFs and determine if these composites could produce any beneficial effects on hydrogen adsorption capacities. Then, MOFs were transformed into carbon by carbonization calcination to achieve carbon composite HNTs. Finally, palladium (Pd) was selected as the noble metal to establish an emerging ternary compound system for the possible promotion of hydrogen adsorption capacities.

## Methods

All of the chemicals were of analytical grade and were used without further purification. Natural halloysites (HNTs) were collected from Hunan, China, and first emulsified with distilled water with a solid–liquid ratio of 1:100 at room temperature, then collected via filtration, washed with deionized water, and dried at 60 °C for 8 h for final use. The emulsifying machine was operated at 6000 r/min. (a) To obtain homogeneous nucleation sites, HNTs were treated with a mixture of nitric acid and sulfuric acid (1:1 *v*/*v*) to introduce carboxylic groups into the HNT surface. Three grams of HNTs was soaked in 750 mL of mixed acid solution for 3 h at different temperatures in an oil bath with stirring, recovered by filtration, washed with deinoized water, and dried at 80 °C to obtain an HNT sample with carboxylic groups. The concentration of the mixed acid solution was selected for 2 and 3 M, while the temperature of the oil bath was selected for 25, 50, and 80 °C. The products were named as H*x*-M (*x* = 2, 3 for the concentration of mixed acid solution, while *M* = 25, 50, and 80 for the reaction temperature). (b) For the samples of Zn series, 1.2 g of zinc nitrate hexahydrate and 0.334 g of terephthalic acid were dissolved in 40 mL of *N*,*N*-dimethylformamide (DMF) during strong stirring for 30 min under atmospheric conditions. Then, 1 g of carboxylic functionalized HNTs was dissolved in the mixed solution and magnetic stirred for another 30 min. 2.2 mL of triethylamine was mixed with 7.8 mL of DMF and was slowly added drop by drop to the solution under agitation. After 30~40 min stirring at room temperature, the white product was filtered off, washed with DMF and ethanol, dried at 80 °C to finally obtain Zn-based MOF composite halloysite (H3-MOFs(Zn)). The powder was transferred into a vertical quartz tube and then calcined at 800 °C for 5 h at a heating rate of 5 °C/min under N_2_ for carbonization. The color of the samples changed from white to black after calcination, and the calcined products were then washed by 3 M hydrochloric acid three times, washed with deionized water, totally dried, and denoted as C-H3-MOFs(Zn); palladium-modified composite (Pd-C-H3-MOFs(Zn)) was synthesized as follows: first, 0.2 g of sodium tetrachloropalladate (Na_2_PdCl_4_) and 0.36 g of polyvinyl pyrrolidone were dissolved in 120 mL of methanol and refluxed for 2 h at 68 °C to form a dark brown solution. Then, 1.5 g of C-H3-MOFs(Zn) was kept in the above solution for 12 h and then rinsed thoroughly with deionized water. Finally, the impregnated sample was reduced by 300 mL of 0.0375 M alkaline solution of hydrazine hydrate (N_2_H_4_·H_2_O) and completely washed with deionized water. (c) For the samples of Al series. 2.84 g of aluminum nitrate nonahydrate and 1.04 g of 1,3,5-benzenetricarboxylic acid were dissolved in 36 mL of ethanol under vigorous stirring for 30 min at room temperature. Then, 2.5 g of carboxylic functionalized HNTs was dissolved in the mixed solution and magnetic stirred for another 30 min. The mixture was transferred to Telfon containers sealed in stainless steel vessels and heated at 120 °C for 1 h. The Al-based MOF composite halloysite (H3-MOFs(Al)) was prepared by drying the wet gel in an oven at 80 °C under stirring; C-H3-MOFs(Al) and Pd-C-H3-MOFs(Al)were synthesized under the same procedures of C-H3-MOFs(Zn) and Pd-C-H3-MOFs(Zn), respectively.

X-ray diffraction (XRD) patterns were recorded on Rigaku D/max 2550 with Cu Kα radiation (*λ* = 0.15406 nm) over a scanning range of 2*θ* = 5°–80° with a step width of 0.02° and at a voltage of 40 kV and a current of 200 mA. Fourier transform infrared (FTIR) absorption spectra of the samples were measured by a Nicolet NEX-US 670 IR spectroscope, analytical grade KBr was used as dispersant, and the range of the spectrum was settled from 400 to 4000 cm^−1^. Transmission electron microscopy (TEM) images were recorded on a JEOL JEM-2100F electron microscope and fitted with an energy dispersive X-ray (EDX) analyzer, at an accelerating voltage of 200 kV. For sample measurements, the powder samples were dispersed in ethanol, as assisted by ultrasonic dispersion for several minutes. The resulting suspension was dripped onto a carbon-coated copper grid and allowed to naturally dry in air. Nitrogen adsorption–desorption isotherms were obtained at −196 °C, using a Micromeritics ASAP 2020 equipment. All the samples were vacuum-dried at 150 °C for 8 h prior to the measurements. The specific surface area (*S*_BET_) was determined from the isotherms by the Brunauer-Emmet-Teller (BET), and the total pore volume was obtained from the maximum amount of nitrogen gas adsorbed at a partial pressure, *p*/*p*_0_, above 0.99. The pore size distribution was calculated by the Barrett-Joyner-Halenda (BJH) method, using the nitrogen adsorption branch of the isotherm.

The hydrogen adsorption isotherms were measured using a static volumetric technique with a specially designed Sieverts apparatus at atmospheric temperature, 25 °C [39]. Prior to the measurements, the apparatus was examined for leakage and calibrated at 25 °C. The dried testing cell with a sample loaded was degassed at 240 °C and at a pressure of no more than 4.0 Pa. Then, hydrogen activation was conducted at a pressure of 1.2 MPa and a temperature of about 140 °C to remove other surface-adsorbed gases. Prior to measurements, the sample was further degassed at 240 °C (pressure <4.0 Pa). After the sample cell cooled down to 25 °C, pure hydrogen at different pressures was injected in the test system to measure the hydrogen adsorption capacity.

## Results and Discussion

**Fig. 1:**
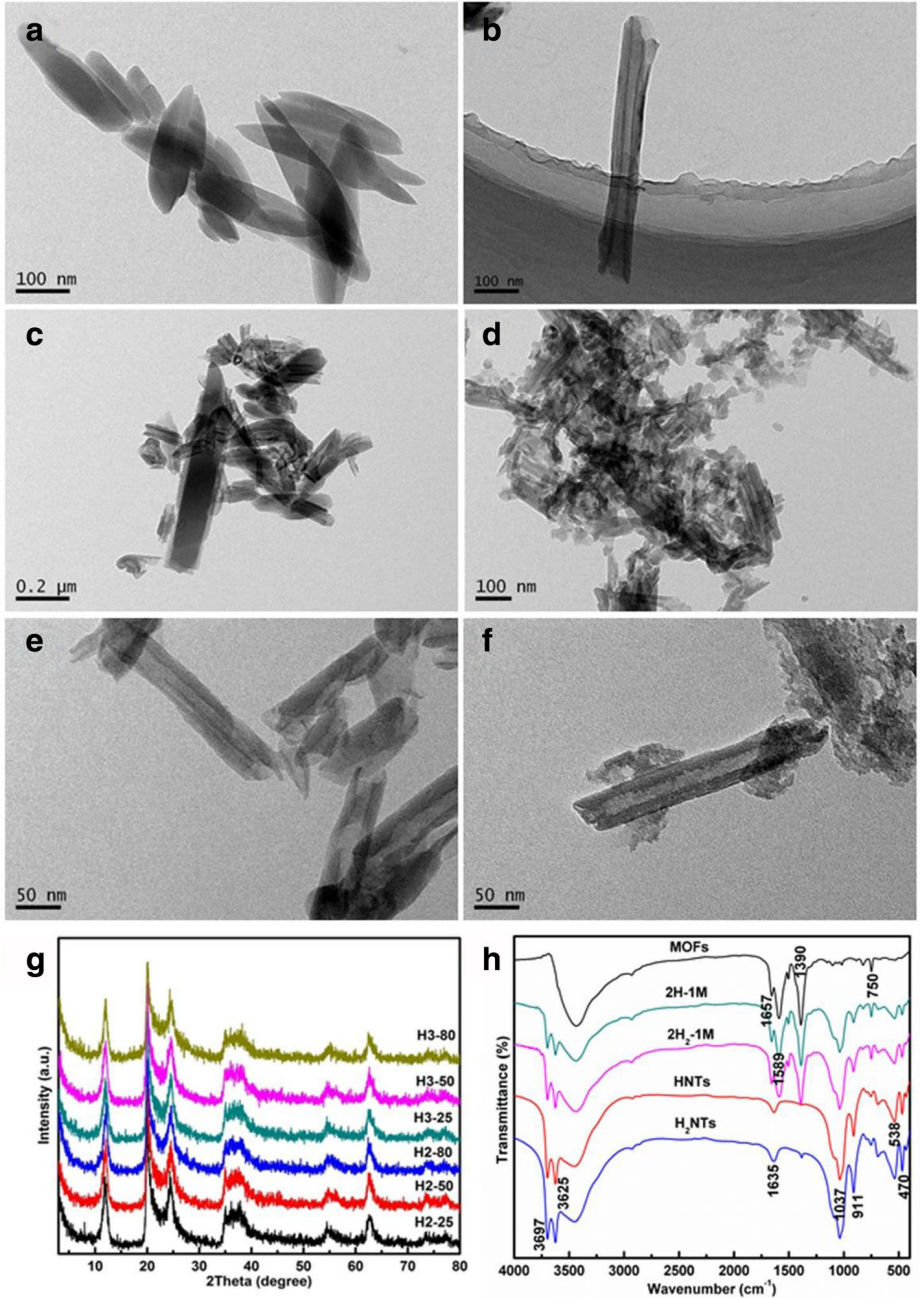
TEM image of **a** MOFs-Zn, **b** HNT, **c** 2H-1M, **d** 2H_2_-1M, **e** H2-25, and **f** H3-80 samples. **g** XRD patterns of acid-treated HNTs under different conditions. **h** FTIR spectra of MOFs-Zn, HNTs, H_2_NTs, and the compounds

The Zn-based MOF (MOFs-Zn) sample features large sheet morphology with an average length of 0.2~0.4 μm and a width of 100~200 nm (Fig. 1a), while the HNT sample features cylindrical hollow tubes of 0.5 μm in length, with an external diameter of 30~75 nm and an internal diameter of 10~30 nm (Fig. 1b). Both the internal wall and outer wall of HNTs are smooth. MOFs feature a larger size than HNTs, and the connection between HNTs and MOFs in the 2H-1M sample (the compound of HNTs and MOFs with a mass ratio of 2:1) is not tight enough (Fig. 1c). Research works have indicated that the surface carboxylate functional groups of a substrate could act as nucleation sites to form MOFs by heterogeneous nucleation and crystal growth [40, 41]. TEM image of 2H_2_-1M reveals that small sheets of MOFs were indeed well admixed with acid-treated halloysite (Fig. 1d). It was assumed that the MOF crystals were formed by heteronucleation and crystal growth on the carboxylic groups of halloysite [42]. More surface edges and defects generated after mixed acid treatment, which can bring higher specific surface energy and serve as nucleation sites for composites [43, 44]. The H2-25 sample keeps the cylindrical hollow tubes of HNTs with an average length of 0.5 μm and an external diameter of 30~75 nm, but the internal diameter increases to 15~30 nm (Fig. 1e). As the acid concentration and temperature increase, the internal diameter of H3-80 increases more, and a lot of small pores formed in the inner wall. The outer wall of H3-80 retained the same smoothness of HNTs, while the inner wall became rough (Fig. 1f). It was assumed that acid treatment started from the inner of tubes and Al ions were removed in the skeleton, which triggered damage on the microstructure of HNTs [45]. XRD patterns of the acid-treated HNT samples under different conditions are presented in Fig. 1g. The acid-treated HNT samples featured comparable XRD diffraction peaks as HNTs, which were indexed to halloysite (JCPDS No. 29-1487). Carboxylic groups could generate by the acid treatment and the basic structures of HNTs remain, judging both from the XRD and FTIR results. However, the intensity of diffraction peaks decreased after acid treatment, suggesting the microstructure of HNTs has been damaged due to the hot-acid corrosion. The sample of H3-80 features the lowest peak intensity as the highest acid concentration and temperature among these samples. HNTs were treated with a mixture of nitric acid and sulfuric acid to introduce carboxylic groups (marked as H_2_NTs). 2H_2_-1M was the compound of H_2_NTs and MOFs with a mass ratio of 2:1. The incorporation of carboxylate groups was confirmed by the FTIR spectra in Fig. 1h. The characteristic band at 1635 cm^−1^ corresponds to C=O, indicating the carboxylate groups in the sample of acid-treated halloysite. The best carboxylic conditions were confirmed among a series of acid-treated HNT samples.

The BET surface area values of different samples are HNTs (64.5 m^2^/g), H2-25 (65.7 m^2^/g), H2-50 (72.4 m^2^/g), H2-80 (162.9 m^2^/g), H3-25 (70.9 m^2^/g), H3-50 (97.7 m^2^/g), and H3-80 (221.4 m^2^/g). The increased internal diameter and pores in the inner wall promoted the increase of the surface area, which instigated by the removal Al ions during the acid treatment. Meanwhile, the surface energy of damaged wall increased, improving the reaction ability with functional groups of other component. Above all, the H3-80 sample was selected as halloysite matrix for composites.

**Fig. 2:**
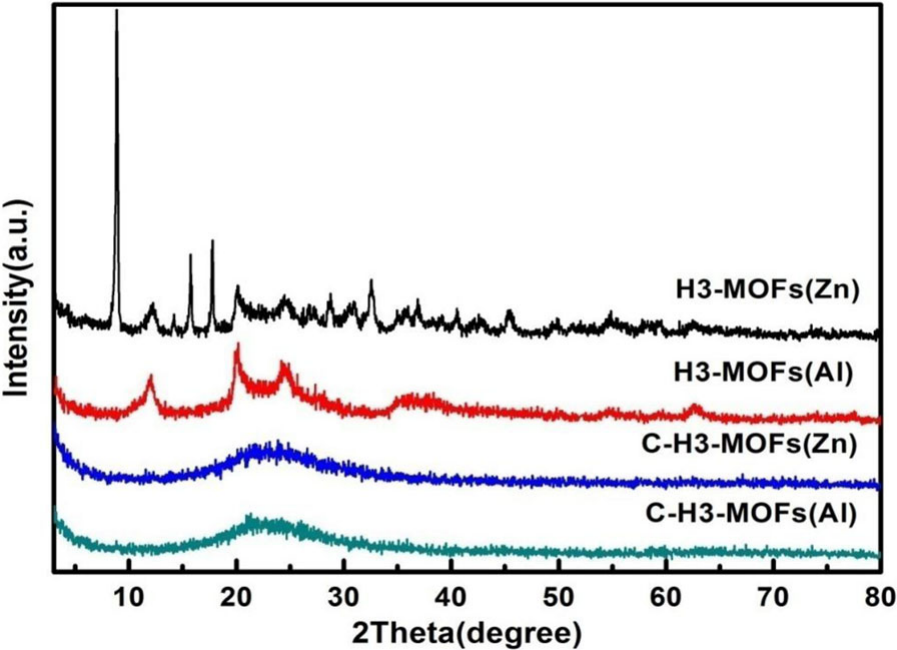
XRD patterns of H3-MOFs(Zn), H3-MOFs(Al), C-H3-MOFs(Zn), and C-H3-MOFs(Al) samples

The H3-MOFs(Zn) sample features both the characteristic diffraction peaks of halloysite and strong diffraction peaks of Zn-based MOFs in Fig. 2, indicating that MOFs of Zn has good crystallinity by precipitation. However, the H3-MOFs(Al) sample just features the characteristic diffraction peaks of halloysite with low intensity, indicating that H3-80 may be coated with amorphous MOFs of Al through a solvothermal method. After carbonization under 800 °C and nitrogen atmosphere, the XRD patterns of both MOF composite H3-80 samples show a broad reflection peak centered at 2*θ* = 20°–25° corresponding the amorphous structure. The presence of halloysite and carbon in the C-H3-MOFs(Zn) and C-H3-MOFs(Al) samples are amorphous caused by calcinations at 800 °C [46].

The BET surface area, average pore size, and pore volume of the MOF composite HNT samples and corresponding carbonized products were listed in Table 1. Heteronucleation and crystal growth of MOFs in the structure of HNTs and structural shrinkage of calcination result in the decrease of the surface area. Nevertheless, the C-H3-MOFs(Zn) and C-H3-MOFs(Al) samples feature a larger BET surface area, average pore size, and pore volume than their precursors, because of the transformation from MOFs to carbon. Organic matters on ignition, leaving spaces to form the pore structure during the carbonized calcination, lead to the promotion of porous characteristics [47].

**Table 1:** Porous parameters of the samples

Samples	BET surface area (m^2^/g)	Average pore size (nm)	Pore volume (cm^3^/g)
C-H3-MOFs(Al)	126.75	8.15	0.2583
H3-MOFs(Al)	104.62	6.35	0.1662
C-H3-MOFs(Zn)	140.82	10.76	0.2986
H3-MOFs(Zn)	121.59	9.04	0.2746

**Fig. 3:**
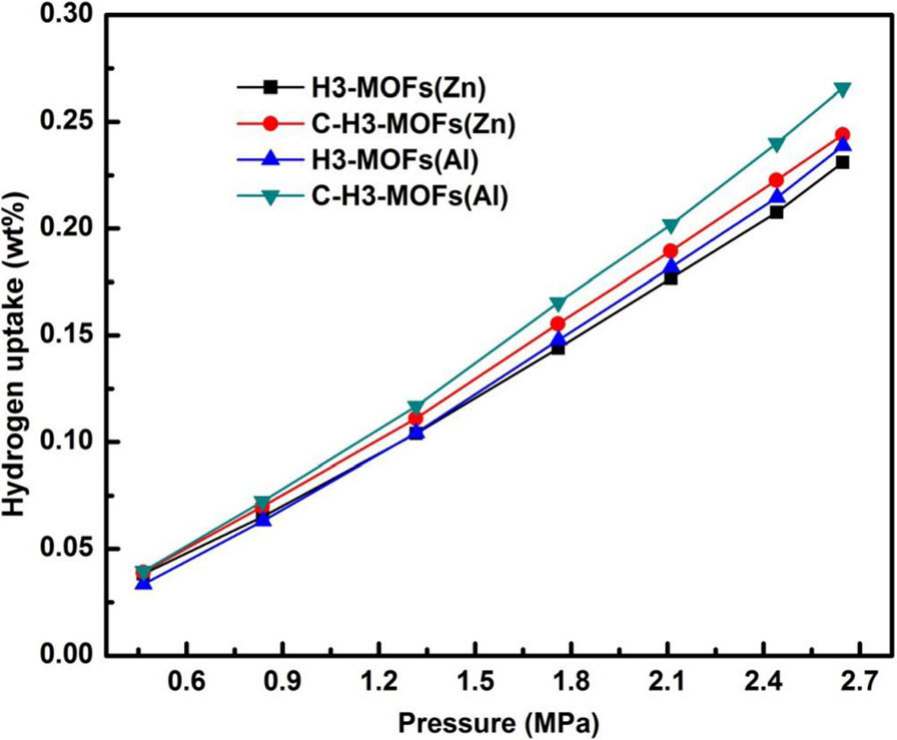
Hydrogen adsorption isotherms of H3-MOFs(Zn), H3-MOFs(Al), C-H3-MOFs(Zn), and C-H3-MOFs(Al) samples

Figure 3 shows the hydrogen adsorption isotherms of MOF composite HNT samples and corresponding carbonized products measured at 25 °C. All the obtained hydrogen adsorption isotherms featured a typical physical adsorption profile [48]: hydrogen adsorption values linearly increase with the increase of pressure. This indicates the hydrogen adsorption on the samples proceeds via a physisorption process [6, 20]. The interlayer spacing of halloysite is a little larger than the molecular kinetic diameter of hydrogen [49], so it allows the hydrogen molecules to enter into the interlayer which leads to a considerable hydrogen adsorption capacity. What is more, the special nanoscale hollow cylinder of halloysite could provide excellent channels to avoid hydrogen blocking compared with other solids [13, 36]. The hydrogen adsorption capacities of H3-MOFs(Zn) and H3-MOFs(Al) are 0.23 and 0.24 wt%, while those of C-H3-MOFs(Zn) and C-H3-MOFs(Al) samples are 0.24 and 0.27 wt%, respectively, derived from the isotherms at 2.65 MPa. Compared with Zn series, Al-based samples both before and after carbonization show higher hydrogen adsorption capacities on account of different selectivity with hydrogen and small pore size [50]. The hydrogen adsorption capacities of C-H3-MOFs(Zn) and C-H3-MOFs(Al) are higher than their precursors. It can be deduced that the promotion of porous characteristics after carbonization could be beneficial towards promoting hydrogen uptake [51].

**Fig. 4:**
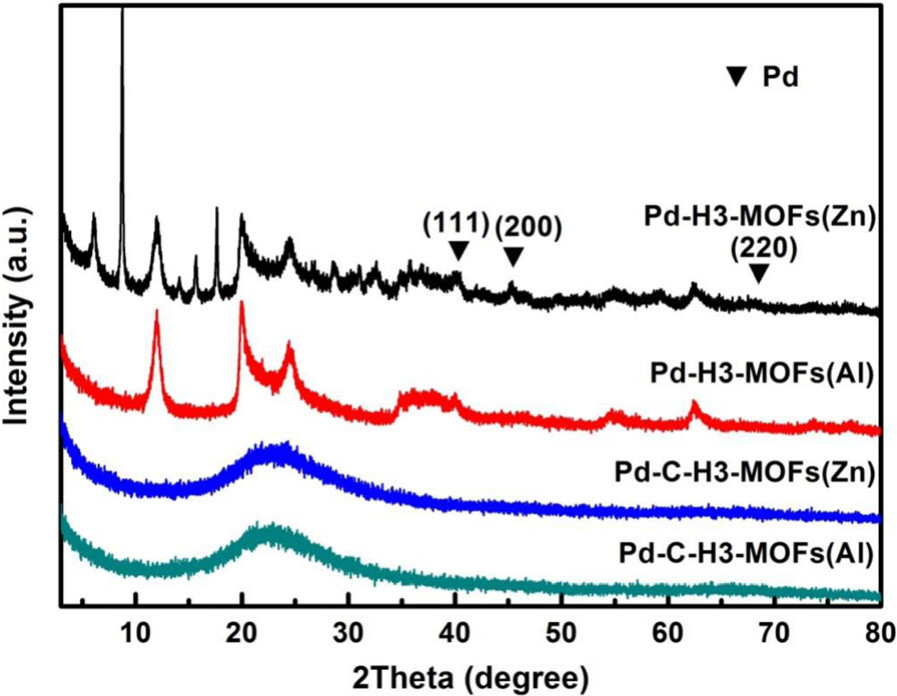
XRD patterns of Pd-H3-MOFs(Zn), Pd-H3-MOFs(Al), Pd-C-H3-MOFs(Zn), and Pd-C-H3-MOFs(Al) samples

Transition metals can be doped to enhance the hydrogen adsorption capacity, and palladium (Pd) is an archetypical hydrogen storage metal [52]. It is possible to enhance hydrogen adsorption capacity through chemisorption or spillover mechanism for the Pd-loaded sample. Herein, Pd was introduced as noble metal for the ternary compound system for the promotion of hydrogen adsorption capacities. Pd-loaded samples feature the same characteristic diffraction peaks as the four precursors, indicating that the phase and basic structure remained intact after Pd incorporation (Fig. 4). The characteristic diffraction peaks of Pd were not obvious, only the peak centered at 2*θ* = 40.1° was observed for Pd-H3-MOFs(Zn) and Pd-H3-MOFs(Al) samples. The low intensity of Pd was due to the low content, complex preparation process of high-temperature calcination, and reduction with hydrazine hydrate solution, which probably leads to the detachment of Pd particles.

**Fig. 5:**
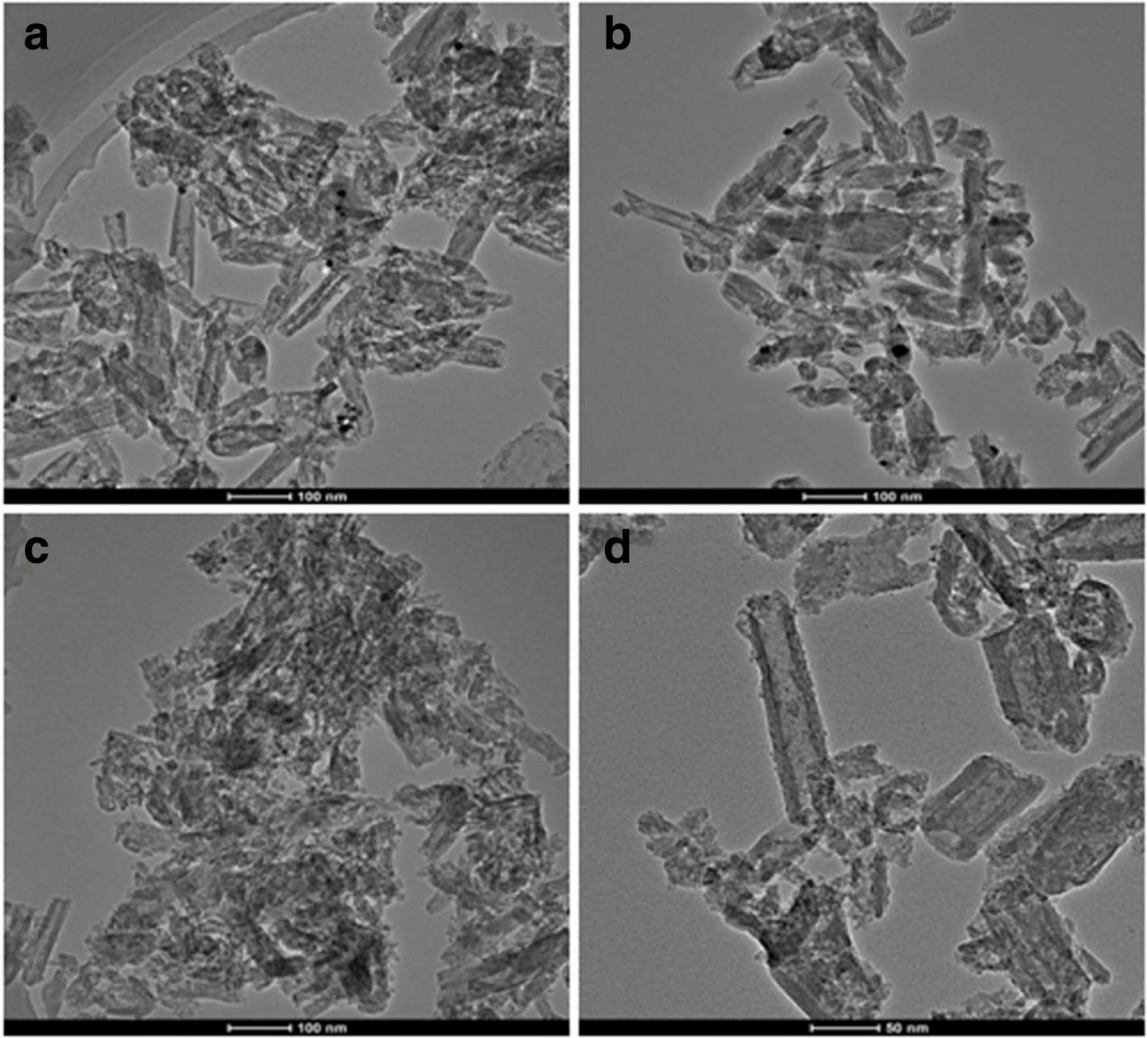
TEM images of **a** Pd-H3-MOFs(Zn), **b** Pd-H3-MOFs(Al), **c** Pd-C-H3-MOFs(Zn), and **d** Pd-C-H3-MOFs(Al) samples

TEM images of Pd-H3-MOFs(Zn) and Pd-H3-MOFs(Al) in Fig. 5a, b display that H3-80 were surrounded by small sheets of MOFs both inside and outside the tubes. Different kinds of MOFs through precipitation and solvothermal methods were well composited with H3-80 and dispersed uniformly. The black spots in the samples of Pd-H3-MOFs(Zn) and Pd-H3-MOFs(Al) are Pd nanoparticles. The dispersibility of Pd particles is worse, and they were reunited during the synthetic process. After carbonized calcination, the small sheets of carbon decreased in size and were well dispersed in the composites. Little amount of Pd particles with a smaller size were in the Pd-C-H3-MOFs(Zn) and Pd-C-H3-MOFs(Al) samples. Some of the Pd particles fell off during the complex preparation process, which results in the unconspicuous XRD patterns and TEM images. The tube morphology of H3-80 had more corrosion structure in the Pd-loaded samples, indicating that reduction of hydrazine hydrate solution could destroy the structure of the tubes of halloysite.

**Fig. 6:**
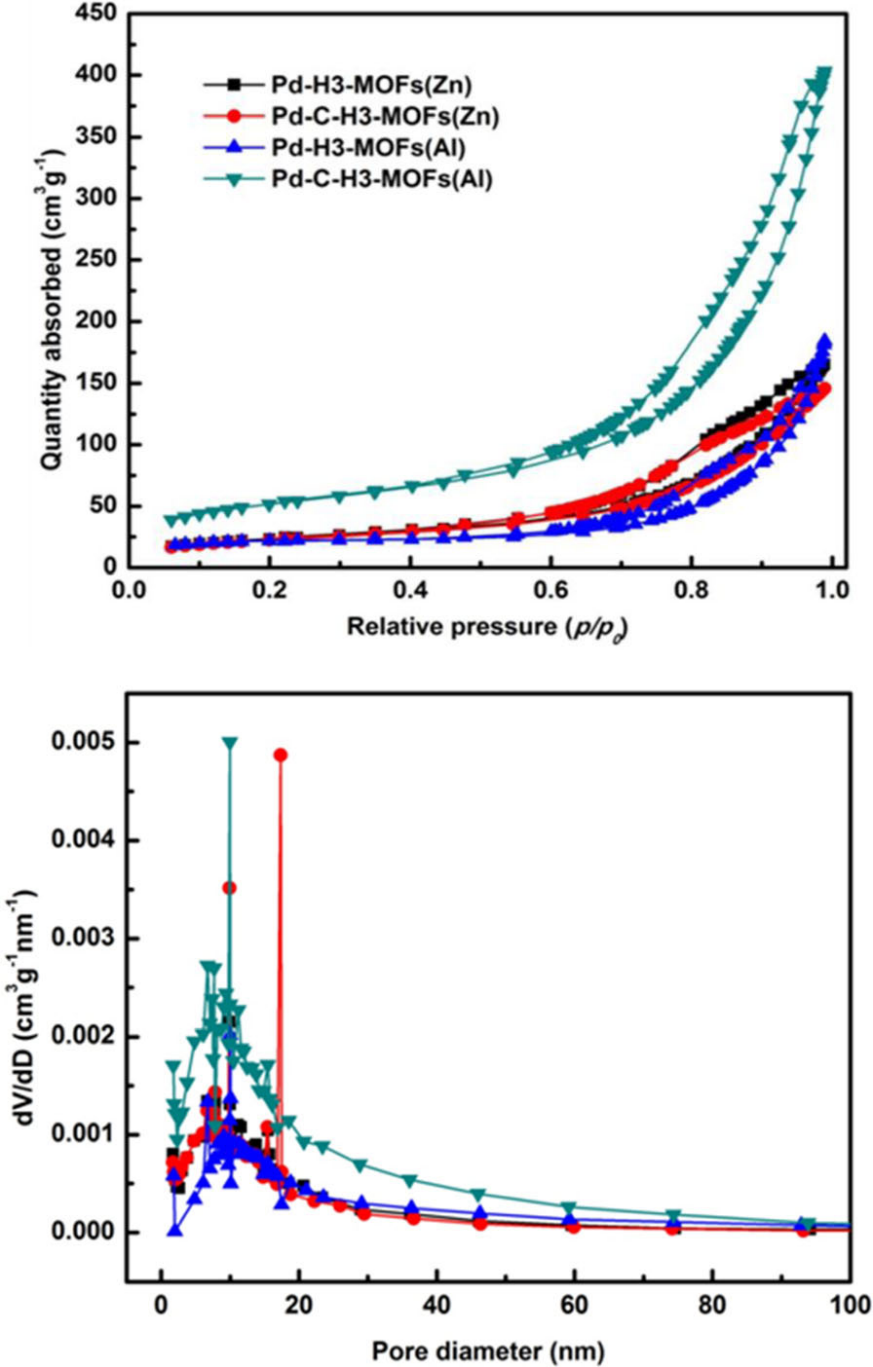
**a** Nitrogen adsorption–desorption isotherms of Pd-H3-MOFs(Zn), Pd-H3-MOFs(Al), Pd-C-H3-MOFs(Zn), and Pd-C-H3-MOFs(Al) samples and **b** their corresponding pore size distributions

Nitrogen adsorption–desorption isotherms of Pd-H3-MOFs(Zn), Pd-H3-MOFs(Al), Pd-C-H3-MOFs(Zn), and Pd-C-H3-MOFs(Al) together with corresponding BJH pore size distributions are shown in Fig. 6. All the isotherms exhibit the same obvious type H3 hysteresis loop in the relative pressure range of 0.7~1.0, indicating that Pd incorporation together with the carbonization do not affect the porous structure. The type H3 hysteresis loop, which does not have adsorption plateau at the high relative pressure section, is an evidence of macropores and cumulate interspaces in the samples [53]. Pd-C-H3-MOFs(Al) presented the most obvious type H3 hysteresis loop attributed to more porous structure.

**Fig. 7:**
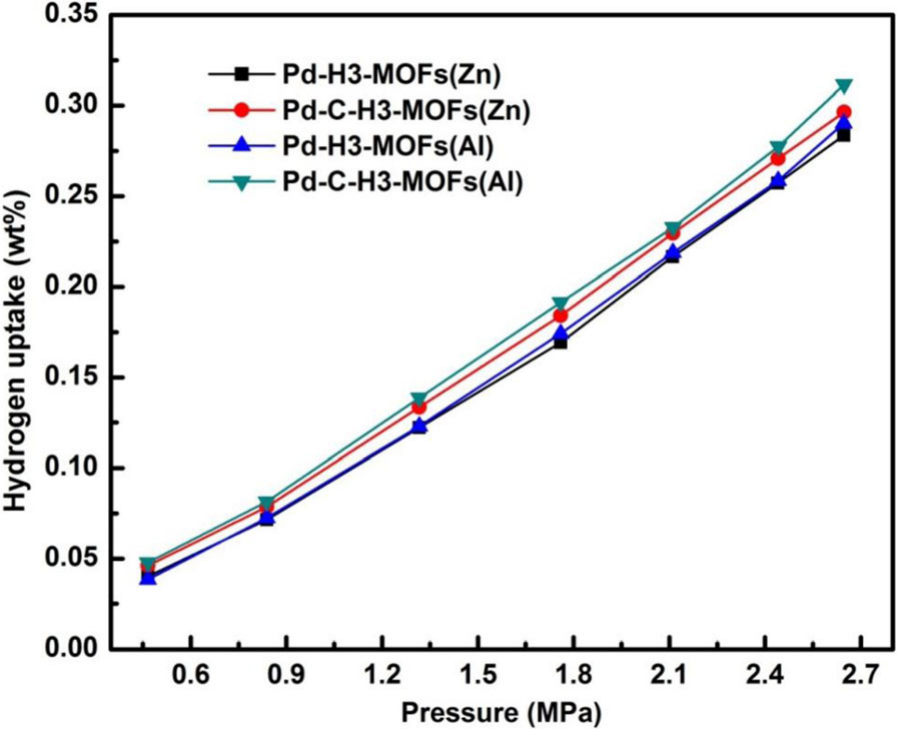
Hydrogen adsorption isotherms of Pd-H3-MOFs(Zn), Pd-H3-MOFs(Al), Pd-C-H3-MOFs(Zn), and Pd-C-H3-MOFs(Al) samples

The hydrogen adsorption isotherms of the Pd-loaded samples measured at 25 °C are shown in Fig. 7. All the Pd-loaded samples show enhanced storage capacity compared to their suitable plain matrix materials. For example, the hydrogen adsorption capacity of Pd-C-H3-MOFs(Al) is 0.32 wt% measured at 25 °C and 2.65 MPa. The promoting hydrogen adsorption capacity could be attributed to the Pd particles [14, 54]. The hydrogen adsorption could be enhanced via chemisorption or spillover [39, 40, 55–57]. Doping with transition metals (such as Pd, Pt, and Ni) is an effective way to improve the hydrogen adsorption capacity via a hydrogen spillover for the physisorption-based materials, which has been experimentally demonstrated [16, 18, 41]. Transition metals have strong ability in storage and dissociation of hydrogen, and Pd is one of the most conventional hydrogen storage metals [42]. Dissociation is assumed to take place on the Pd particles, and atomic and molecule hydrogen spill over to the structure of H3-80, MOFs, and its carbon product [18, 21, 39, 58]. The atomic and molecular hydrogen could be adsorbed within the MCM-41 matrix by physical adsorption.

## Conclusions

Emerging composites of halloysite and MOFs were synthesized by different methods. Then, MOFs were transformed into carbon by carbonized calcination, and Pd was incorporated to establish a ternary compound system for the promotion of hydrogen adsorption capacities. Al-based samples show higher hydrogen adsorption capacities on account of different selectivity with hydrogen of metal and approximate porous characteristics. More pore structures are generated by the thermal reaction from metal–organic frameworks to carbon structure; high specific surface area, uniform pore size, and large pore volume promote the hydrogen adsorption ability of composited tubes. Moreover, dissociation is assumed to take place on the Pd nanoparticles, and atomic and molecule hydrogen spill over to the structures of HNTs, MOFs, and its carbon product for improving the hydrogen adsorption capacity.

## Abbreviations

BET: Brunauer-Emmet-Teller
BJH: Barrett-Joyner-Halenda
DMF: Dimethylformamide
EDX: Energy dispersive X-ray
FTIR: Fourier transform infrared
HNTs: Halloysite nanotubes
MOFs: Metal–organic frameworks
Pd: Palladium
SEM: Scanning electron microscope
TEM: Transmission electron microscopy
XRD: X-ray diffraction
